# Blending group-based psychoeducation with a smartphone intervention for the reduction of depressive symptoms: results of a randomized controlled pilot study

**DOI:** 10.1186/s40814-021-00799-y

**Published:** 2021-02-24

**Authors:** Christian Aljoscha Lukas, Matthias Berking

**Affiliations:** Department of Clinical Psychology and Psychotherapy, Friedrich-Alexander-University, Erlangen-Nuremberg, Germany

**Keywords:** Depression, Approach–avoidance, Smartphone, Intervention, Pilot study

## Abstract

**Background:**

Given their ubiquity and technological facilities, smartphone-based interventions (SBIs) hold potential to support the cost-effective dissemination of evidence-based treatments for depression. As technologically enriched, blended approach–avoidance modification trainings (AAMTs) have recently been shown effective for symptom reduction in various mental health problems, we developed a blended SBI combining group-based psychoeducation and 14 days of app training utilizing principles from AAMT to reduce depressive symptoms.

**Methods:**

In this pilot trial, *N* = 16 individuals with heightened depression scores were randomized to either an intervention group using the mentalis Phoenix app or a wait list control condition. As outcomes, we descriptively explored usability of the app, engagement with the intervention, and possible reductions of depressive symptoms.

**Results:**

Data analyses suggest that the SBI tested in this pilot trial possesses high usability, is frequently engaged with, and reduces depressive symptoms in participants in the intervention group when compared to wait list controls.

**Conclusions:**

This pilot study provides preliminary evidence that an SBI utilizing AAMT can reduce depressive symptoms. Future studies should replicate these findings using larger samples and disentangle possible mechanisms of change.

**Trial registration:**

DRKS-ID: DRKS00021613 (retrospectively registered).

**Supplementary Information:**

The online version contains supplementary material available at 10.1186/s40814-021-00799-y.

## Key messages regarding feasibility

- What uncertainties existed regarding the feasibility?
- Major uncertainties included (a) usability of the prototype app for the utilization of the therapy, (b) engagement with the intervention, and (c) possible effects of the intervention on depressive symptoms.
- What are the key feasibility findings?
- Rating of usability was high, engagement with the intervention was frequent, and depressive symptoms were reduced in the intervention group. However, a major problem not predicted before conducting the study included technical difficulties occurring after randomization, which caused high dropout rates.
- What are the implications of the feasibility findings for the design of the main study?

The findings of this pilot trial indicate that the intervention under investigation holds the potential for the reduction of depressive symptoms. Given the technical problems observed during the study, we are planning to enhance server capacity and to conduct several beta tests of the software before starting the main study.

## Background

Given the high prevalence rates for depression and the negative consequences for afflicted individuals, psychotherapeutic research has focused on the development of effective treatments for this mental disorder over the last decades [37]. Despite numerous studies providing evidence for the effectiveness of psychological treatments targeting depression [10], only 40% of afflicted individuals respond (fully or partially) to psychotherapy [24]. Furthermore, some studies have demonstrated that patients undergoing psychotherapy only experience a partial remission of depressive symptoms [29]. Another limitation is that a large number of afflicted individuals do not receive evidence-based treatment for their depressive symptoms [37]. Thus, there is ongoing demand for the development of further evidence-based treatments that are easy to disseminate.

In his cognitive theory of depression, Aaron T. Beck postulates that biased processing of information plays an important role for the development and maintenance of depression [3]. Following his theory, depressed individuals—when compared to nondepressed individuals—selectively occupy themselves with negative stimuli and information and have difficulty disengaging from such material. This cognitive bias then substantiates sad mood and can lead to a depressive episode in the long term [5]. Empirical evidence for this theory can be found in a meta-analysis by Peckham et al. [30], showing that depressed individuals turn more strongly towards negative information and less strongly to positive information when compared with nondepressed individuals.

On the basis of this theory, cognitive bias modification trainings (CBM) that are conducted in computerized procedures have been discussed as potentially promising for the systematic modification of biased processes. This interest in computerized CBM can be explained by the low-threshold use, the potential for using modern technologies for the administration of training, and the cost-effectiveness when compared with traditional therapeutic interventions [38]. Another advantage of CBM is the focus on implicit processes that, when compared to explicit cognitive techniques frequently used in traditional psychotherapy, initially does not require elaborated patient insight [15].

The effectiveness of CBM trainings for depression has been shown in several studies. A study by Beevers et al. [5] demonstrated that a CBM intervention for depression successfully modified an existing dysfunctional cognitive bias and led to a reduction in depressive symptoms. Further evidence for the effectiveness of CBM programs for depression comes from a study by Smith et al. [33] showing that a CBM training targeting biased interpretations reduces the interpretation of stimuli as hostile when compared with a control group (*d* = 0.53). However, meta-analytic results on the effectiveness of CBM for depression are more inconsistent. A meta-analysis by Hallion and Ruscio [16] reports a medium effect (*g* = 0.49) on changes in cognitive biases, but another meta-analysis by Cristea et al. [9] shows that CBM trainings only yield small effects (*g* = 0.24) on clinical depression. This meta-analysis also demonstrated that effects are reduced to a minimum (*g* = 0.04) after adjusting results for publication bias.

More consistent results for the use of a sub-paradigm of CBM—the approach–avoidance modification training (AAMT) that focuses on the modification of approach–avoidance biases—come from research on substance use. In AAMT, bias modification is achieved using a modified approach–avoidance task [21] which is usually administered on a computer. In a study by Wiers et al. [36], testing the AAMT, 214 alcohol-dependent patients received either a combination of 3 months of inpatient cognitive behavioral therapy (CBT) with computer-based AAMT or CBT-only. In the computer-based AAMT, participants were asked to pull a joystick (approach movement) upon the demonstration of abstinence-related stimuli and to push a joystick (avoidance movement) upon seeing alcohol-related stimuli. Results from this study show that 4 sessions of AAMT modified an approach bias to an avoidance bias towards alcohol and effectively reduced relapse rate at 12 months of follow-up by 13% when compared with CBT-only controls. Results from this study were replicated in a study administering 12 sessions of AAMT, leading to a 10% reduction of relapse at 12 months of follow-up [11]. Given the demonstrated importance of approach–avoidance biases for the development and maintenance of depression [32], computerized AAMT has also been evaluated in the domain of depression. In a study by Vrijsen [35], computerized AAMT has been added to treatment-as-usual in a sample of depressed patients and was shown to successfully reduce depressive symptoms when compared with a sham control condition. Furthermore, a study by Becker et al. [4] tested AAMT as an adjunct to inpatient treatment and found a reduction of depressive symptoms compared with a sham control group.

With regard to the promising results found in research on AAMT and the ubiquity of mobile devices in the general population [34] and in patients with mental disorders [2], we developed several smartphone-based interventions (SBIs) utilizing AAMT principles in combination with techniques from CBT. In these studies, 14 days of smartphone AAMT were blended with face-to-face counseling sessions either in individual or in group settings and effectively reduced symptoms of procrastination (*d* = 0.84), body dissatisfaction (*d* = 0.62), eating disorders (*d* = 0.46), and alexithymia (*d* = 0.97) when compared with heterogeneous control conditions [22, 25, 26]. Based on the promising findings of blended SBIs for other mental health problems and meta-analytic results [14] showing that SBIs can successfully reduce depressive symptoms when compared with wait list control conditions (*g* = 0.38), we developed the SBI mentalis Phoenix (MT-Phoenix) targeting depression.

Given the need for high-quality research when assessing possible effects of psychotherapeutic treatments, we conducted a pilot study before implementing a large randomized controlled trial. As there are no study results available that provide information on SBIs using AAMT for depression, the objectives of this pilot study were to test the usability of the app MT-Phoenix and the engagement of depressed participants with this new intervention. Furthermore, we explored whether MT-Phoenix can reduce depressive symptoms.

## Methods

### Study design

To (a) generate first data on acceptance, usability, and intervention engagement and (b) test whether MT-Phoenix has the potential to effectively reduce depressive symptoms, we conducted a pilot study with two groups. The intervention group received a combination of 14 days of app training and a face-to-face psychoeducative group counseling session prior to the training. The control group was a wait list control group. In this pilot trial, participants were randomly assigned to these two conditions (intervention group or wait list control group). Data were assessed at pretreatment, posttreatment, and 1-month follow-up. All study procedures complied with the human research guidelines of the Helsinki Protocol and were approved by the ethics committee of the German Psychological Society. For the CONSORT Checklist, see Additional file 1.

### Participants

**Fig. 1:**
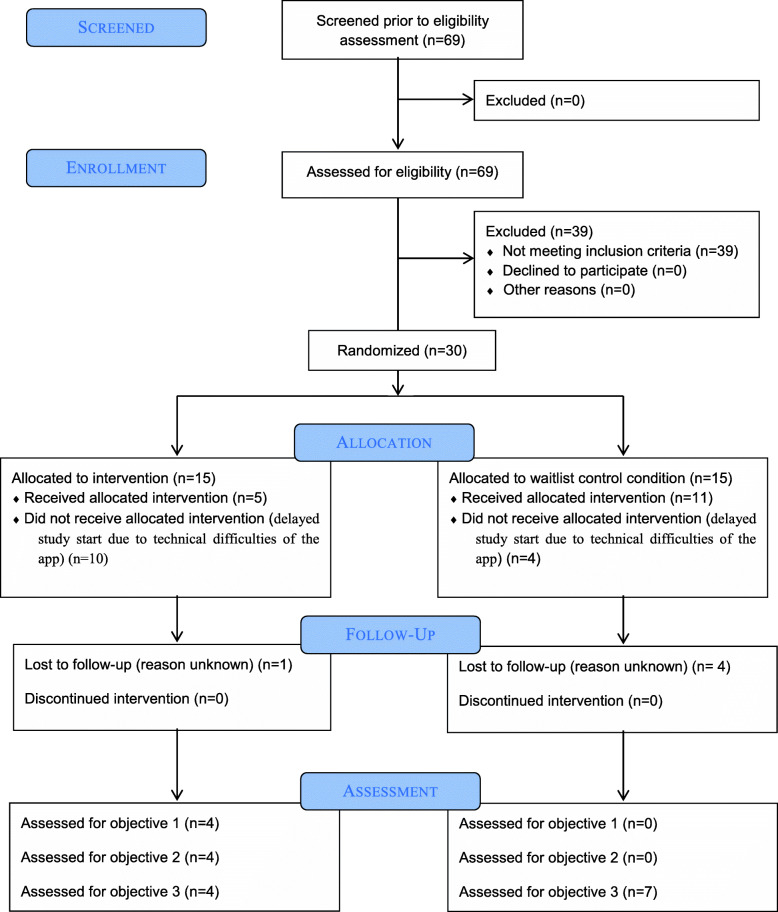
CONSORT flow diagram

Participants were recruited via Facebook (e.g., university and local groups) and several recruitment posters on campus. Inclusion criteria for the study were (a) heightened depression scores with values ≥ 10 on the Patient Health Questionnaire-9 (PHQ-9 [23];), (b) access to a smartphone using Android operating system (version 4 or above), (c) age 18 or above, and (d) informed consent. Participants who reported current suicidal ideations were excluded from study participation and delegated for further clarification to psychotherapists at the department’s outpatient center. Following the recommendations by Browne [8] and Julious [20] and experience with regard to participant attrition in studies on digital interventions targeting mental health problems, a total of 69 participants were screened, so that 30 participants meeting all the inclusion criteria and none of the exclusion criteria could be considered for study inclusion. After returning a signed copy of the informed consent form, participants were randomly assigned to either the intervention (*n* = 15) or the waitlist control condition (*n* = 15). A simple randomization was used and conducted by three master’s degree students (via https://www.randomizer.org/). Due to technical difficulties with the app that caused a delay of several weeks, of the number of participants participating in the study dropped to *N* = 16. Thus, at the beginning of the study, the sample consisted of 16 participants (intervention group: *n* = 5; waitlist control group: *n* = 11). Figure 1 illustrates the flow of participants throughout the study. Participants were predominately female (81%) with an average age of 24.69 years (SD = 4.47). With regard to education, all participants reported to have completed 12 years of education or more. Four individuals in the wait list control group reported a diagnosed psychiatric disorder in the past (i.e., major depressive disorder, moderately recurrent depressive disorder, moderately recurrent depressive disorder and comorbid borderline personality disorder, and severe depressive episode without psychotic symptoms). Furthermore, five participants in the wait list control condition received psychotherapy in the past and one participant reported to be in therapeutic treatment during the study. For sociodemographics of study participants, see Table 1.

**Table 1:** Sociodemographic data

	Intervention group (*n* = 5)	Control group (*n* = 11)
**Age** (years) *M* (SD)	25.60 (3.65)	24.27 (4.90)
**Gender** *n* (%) female	3 (60)	10 (90)
**Education** *n* (%)		
High school diploma	4 (83)	9 (81)
University degree	1 (17)	2 (19)
**Diagnosed mental disorder** *n* (%)		
No	4 (80)	7 (64)
Yes	0 (0)	4 (36)
Declined to answer	1 (20)	0 (0)
**Psychotherapy (past)** *n* (%)		
No	4 (80)	6 (55)
Yes	0 (0)	5 (45)
Declined to answer	1 (20)	0 (0)
**Psychotherapy (currently)** *n* (%)		
No	4 (80)	10 (90)
Yes	0 (0)	1 (9)
Declined to answer	1 (20)	0 (0)

### Intervention

#### Psychoeducation

The face-to-face psychoeducation sessions were conducted in groups of five participants, lasted 45 min, and consisted of three parts: (1) Understanding my depression, (2) Introduction to the functionalities of MT-Phoenix, and (3) Creating customized stimulus material for the AAMT. In (1) *Information on depression*, for a better understanding of depressive symptoms and to promote destigmatization, participants received general information about depressive symptoms and were then invited to develop an individual model of the emergence of depression based on the biopsychosocial model [12]. Finally, participants were asked to identify factors responsible for the maintenance of depressive symptoms based on Beck’s cognitive triad [3]. In (2) *Introduction to MT-Phoenix*, participants were introduced to the app and instructed in its handling by watching an instructor showing its key functions. In (3) *Stimulus material*, the last step of the session, participants created customized stimuli by devising 20 dysfunctional (e.g., “I am a failure”) and 20 functional (e.g., “It’s okay not to be okay”) stimuli. In the app, 40 customized and 40 standardized stimuli were used for the AAMT. The standardized stimuli were created by a graduate psychologist and a professor in clinical psychology.

#### Smartphone app

In the 1.0 version of MT-Phoenix used in this pilot study, symptom reduction is intended by reducing dysfunctional cognitions and behavior that contribute to the maintenance of depressive symptoms and increasing functional cognitions and behavior that can help reduce depressive symptoms. To this end, the app displays stimulus material (statements, pictures, combination of both) that users are asked to either swipe away (for depressogenic stimuli) or to pull towards themselves (for functional stimuli) in a training session of about 5 min. Based on self-consistency theories [6, 13], it can be expected that the AAMT in MT-Phoenix leads to negative attitudes towards dysfunctional cognitions and behavior and to a positive attitude towards functional cognitions and behavior. To systematically increase the frequency of functional tendencies and promote the generalization of learned reactions into everyday life [31], MT-Phoenix reinforces the user by providing gamified operant conditioning giving feedback upon correct (smiling emoji and the word “Correct!”) and incorrect reactions (frowning emoji, the words “That’s wrong!”, and a short vibration of the smartphone). Following the psychoeducation session, participants downloaded the app from the Google Play Store and were instructed to complete one training session per day over the intervention period of 14 days. The app was available for Android devices only (version 4 or higher).

### Measures

Acceptance and usability of the application was assessed with the System Usability Scale (SUS [7];) at post assessment. The SUS is a widely used and standardized 10-item questionnaire for evaluating the usability and user-friendliness of a system (e.g., software, websites) on a five-point Likert scale ranging from 0 to 4. Scores can range from 0 to 40 and are then multiplied by 2.5 to convert the original score to 0 to 100. Higher scores indicate a higher usability, scores above 80 are considered “excellent”. Internal consistency of the SUS has been shown to be good with alpha scores ranging from .85 to .92 [1].

For the assessment of depressive symptoms we used the PHQ-9 questionnaire [23]. On a Likert-type scale ranging from 0 to 3, the PHQ-9 evaluates depressive symptoms during the last 14 days. Sum scores can range from 0 (absence of depressive symptoms) to 27 (severe depressive symptoms). Internal consistency has been demonstrated as good with alpha scores ranging from .86 to .89 [23]. The cutoff used for the PHQ-9 in this study (≥ 10) has a sensitivity of 88% in detecting depressive symptoms [27].

### Feasibility outcomes

To evaluate success of feasibility, the following criteria were determined:

1. Participants rate usability higher than average (> 68).
2. On average, participants engage with the intervention at least 3.5 times a week, which represents 50% of the recommended time.

### Statistical analyses

For the evaluation of the feasibility criteria under investigation in this study, we drew on the descriptive statistics from the SUS to test acceptance and usability of MT-Phoenix and analyzed data on participants’ app usage to test engagement with the intervention. For the exploration of the hypothesis that the intervention would significantly reduce depressive symptoms in the intervention group when compared with the wait list control condition, we first imputed missing data from the five participants that dropped out from assessment and utilized an intention-to-treat approach using multiple imputation (MI) with the help of a Markov Chain Monte Carlo MI algorithm. As sample sizes were small, we refrained from computing test statistics such as mixed ANOVAs that are usually used for analyzing data in similar study designs. Instead, we provide descriptive statistics to consider possible intervention effects. All statistical analyses were conducted using SPSS 25.

## Results

### Feasibility evaluation

Regarding the evaluation of the acceptance and usability of MT-Phoenix, participants reported a high degree of satisfaction (*M* = 91.25, SD = 2.06) on the SUS. When assessing engagement with the intervention from server data, results revealed that participants used the app on average for 8.2 days (SD = 3.56, range = 3–11) for an average of 41.31 min (SD = 39.86, range = 8.8–121.87). Participants completed an average of 9.4 training sessions (SD = 4.28, range = 3–13).

### Exploratory analysis

Descriptive data on possible effects of the SBI on depressive symptoms suggest that participants in the intervention group experienced greater reduction in depressive symptoms than did the wait list control group. Descriptive data are displayed in Table 2.

**Table 2:** Means and standard deviations

Outcome	Group	Baseline *M* (SD)	Posttest *M* (SD)	Follow-up *M (*SD*)*
PHQ-9	Intervention	14.20 (4.09)	8.35 (5.32)	8.80 (2.38)
	Wait list control	15.55 (2.42)	15.87 (4.14)	16.00 (4.37)

Note: *PHQ-9* Patient Health Questionnaire-9

## Conclusions

This pilot study evaluated a blended intervention combining a face-to-face group-based psychoeducation session and 14 days of training with the MT-Phoenix app aiming to reduce depressive symptoms. To draw first conclusions about this intervention, we examined data on participants’ acceptance and rating of usability, engagement rates with MT-Phoenix, and the possible efficacy of the blended intervention with regard to the reduction of depressive symptoms. Results show that the app received high ratings with regard to acceptance and usability. Engagement rates indicate that participants used the app frequently (average of 8.2 days out of 14 possible days). However, as participants were instructed to train with the app every day, engagement rates were not as high as recommended in this study. Nevertheless, both determined feasibility criteria were met; hence, the feasibility of this intervention can be considered successful. With regard to the reduction of depressive symptoms, the descriptive data shows a reduction in the intervention group over the intervention period and no reductions for wait list controls. These reductions were sustained at 1-month follow-up.

Study results provide preliminary evidence for a blended intervention combining face-to-face elements and an SBI utilizing AAMT for the reduction of depressive symptoms and could have important theoretical and practical implications if replicated in future studies. First, findings show that the systematic modification of approach–avoidance tendencies can lead to reductions of depressive symptoms. Even if the AAMT represented only one part of the intervention, these findings make an important contribution to existing literature on the effectiveness of such paradigms for treatment of several mental disorders. Second, they deliver further evidence for the potential efficacy of SBIs that offer interventions beyond the use of traditional CBT techniques for the reduction of depressive symptoms. Third, findings indicate that even brief digitalized psychotherapeutic interventions can lead to changes in depressive symptoms. Finally, this study points that the use of gamified intervention components for treating mental disorders can be transferred from computer-based interventions [17, 28] to SBIs.

Although promising, the presented results are preliminary and should be interpreted with caution. Limitations that prelude the generalization of results include (1) the use of a small and nonclinical sample, (2) high dropout rates before the start of the study, (3) differences with regard to mental health diagnoses and ongoing psychotherapy between the intervention and the wait list control group, (4) the use of only self-report measures, and (5) the absence of experimental manipulation of potential change mechanisms. First, due to the small sample size used in this pilot study, results should be interpreted with caution as they are limited in generalizability [18, 19]. Future studies should use larger samples to gather more robust results on possible effects of this intervention. Second, the high dropout that occurred before baseline assessment can be attributed to two possible reasons: (a) technical problems during the deployment process of the app caused a delay of the study of 6 weeks, and (b) there were problems concerning the face-to-face format of the psychoeducation. Regarding the latter, an additional survey after the end of the study revealed that participants felt uncomfortable with the group format for the psychoeducation session. Thus, beyond the aforementioned delay, it can be assumed that dropout may also have occurred due to fear of stigmatization. In the future, studies should also test whether an automated and therefore anonymous psychoeducational format (administered via app) that is blended with smartphone-based AAMT may help to solve issues related to study dropout. Third, participants in both groups differed with regard to prior mental health diagnoses as participants in the wait list control group reported were more frequently diagnosed than participants in the intervention group. These differences may have contributed partially to the examined intervention effects. Fourth, the use of only self-report measures is another limitation. Future studies should complement self-report assessments with observer-based, interviewer-based, biological or additional experimental measures. Finally, the study design does not allow a conclusion with regard to the discrete change mechanism responsible for the treatment effect observed in this study. Hence, it is unclear to what extent the elements specific to this study such as the face-to-face psychoeducation session, the SBI-based AAMT, the combination of both, the use of customized stimulus material, or the operant conditioning paradigm included in the app were responsible for the reported improvements. Therefore, future research should investigate potential mechanisms of change systematically using experimental manipulations.

## Supplementary Information

**Additional file 1.** CONSORT Checklist.

## Data Availability

The datasets used and analyzed during the current study are available from the corresponding author on reasonable request.

## Abbreviations

SBI: Smartphone-based intervention
AAMT: Approach–avoidance modification trainings
CBM: Cognitive bias modification
CBT: Cognitive behavioral therapy
